# A molecular study of the genus *Spelaeomyia* (Diptera: Phlebotominae) with description of the male of *Spelaeomyia moucheti*

**DOI:** 10.1186/s13071-016-1656-5

**Published:** 2016-06-29

**Authors:** Nil Rahola, Leila Hadj Henni, Judicael Obame, Diego Ayala, Boris Kevin Makanga, Véronique Lehrter, Arezki Izri, Christophe Paupy, Jérôme Depaquit

**Affiliations:** Unité MIVEGEC, UMR 224-5290 IRD-CNRS-UM, Centre IRD de Montpellier, BP 64501, 34394 Montpellier, France; Centre International de Recherches Médicales de Franceville (CIRMF), BP 769 Franceville, Gabon; Université de Reims Champagne Ardenne, ANSES, SFR Cap santé, EA 4688-USC “Transmission vectorielle et épidémiosurveillance de maladies parasitaires (VECPAR)”, Reims, France; Parasitologie, Hôpital Avicenne, Université de Paris 13, Paris, France & unité des virus émergents, UMR 190 (IRD-AMU – EHESP), Marseille, France

**Keywords:** Sand fly, *Spelaeomyia*, Morphology, Phylogeny, Africa

## Abstract

**Background:**

The genus *Spelaeomyia* includes four African species considered as being cavernicolous: *Spelaeomyia darlingi*, *Spelaeomyia mirabilis*, *Spelaeomyia emilii* and *Spelaeomyia moucheti*. Despite a potential role in *Leishmania major* leishmaniasis transmission in Mali, no molecular studies and only few morphological studies have addressed relationships between species of *Spelaeomyia*.

**Methods:**

Specimens of *Sa. moucheti* were collected in two different sites in Gabon. *Spelaeomyia emilii* and *Sa. darlingi* specimens came from Gabon and Mali. Specimens of *Sa. mirabilis* were collected in the Democratic Republic of Congo and Gabon. All specimens were caught using CDC miniature light traps, then dissected, both heads and genitalia were kept for morphological analysis and the rest of the bodies were kept for molecular processing and analyses.

**Results:**

Some unidentified males are associated to *Sa. moucheti* females using molecular tools and are described for the first time. A new morphological feature is observed on the spermathecae of the female and new drawings are provided. For the first time a phylogenetic analysis is carried out on rDNA and mtDNA markers and it shows that *Sa. moucheti* is the sister species of *Sa. mirabilis*.

**Conclusions:**

*Spelaeomyia moucheti* is the sister species of *Sa. mirabilis*. This result is in agreement with the sharing of morphological characters between these closely related species. Moreover, these two species are not as cavernicolous as literature previously indicated. They were caught in open rainforest in Gabon.

## Background

The genus *Spelaeomyia* Theodor was created as a subgenus of *Sergentomyia* França & Parrot [1] and defined as a strictly cavernicolous group of phlebotomine sand flies. It was erected at the generic level by Artemiev during a revision of the classification of Phlebotominae [2] and then commonly considered by systematicians at this taxonomic level [3–6].

The genus includes four species, all recorded in sub-Saharan continental Africa [7]: *Spelaeomyia darlingi* (Parrot & Wanson, 1939) designated as the type-species of the genus, *Sa. mirabilis* (Kirk & Lewis, 1954), *Sa. emilii* (Vattier-Bernard, 1966) and *Sa. moucheti* (Vattier-Bernard & Abonnenc, 1967). This genus has been under-studied despite the possible role of its species in *Leishmania major* leishmaniasis transmission in Mali [8] possibly due to their supposed cavernicolous ecology and trophic specialization on bats.

Our sampling includes all the known species of the genus. In order to assess the taxonomic relationships among members of the genus *Spelaeomyia*, we carried out a molecular phylogeny based on ribosomal and mitochondrial markers.

## Methods

### Sand fly sampling

The different specimens used in our study come from various sampling projects. We had access to *Sa. mirabilis* during a project of molecular systematics. An epidemiological study in Mali [9] allowed us to collect some *Sa. darlingi*. Furthermore, it was during a recent faunistic research project aimed at the study of the Culicinae fauna of Gabon that we caught some *Sa. mirabilis*, *Sa. emilii*, females of *Sa. moucheti* and unknown males. All specimens were collected using CDC Miniature Light Trap incandescent lights (John W. Hock Company, Gainesville, Florida, USA) from dusk to dawn outside caves and on a 24-h basis inside caves.

### Preparation of samples for morphological study

The sand flies were preserved in 100 % ethanol and then the whole carcasses (thoraces, wings and legs) were kept for molecular studies, while heads and genitalia were dissected and mounted in Euparal after different successive baths: 2 h in 10 % potassium hydroxide; 2 h in distillated water; 10 h in a Marc-André solution [7]; 10 h in distillated water; 20 min in 70 % ethanol; 20 min in 90 % ethanol; 20 min in 100 % ethanol; and 10 h in a beech wood solution. The spermathecae of the *Sa. moucheti* females were first removed from the abdomen and then observed with a simple clearing of heated Marc-André solution (90 °C).

Specimens were observed using BX 53 (Olympus, Japan) and Leica DM2000 microscopes. Measurements (in micrometers unless otherwise indicated) were taken using the Stream motion software (Olympus, Japan) and a video camera (Leica and Olympus, respectively) connected to the microscope. The figures were drawn by hand using a drawing tube connected to the microscope.

### Sequencing

In the manuscript, we call “Cyt b” a part of the cytochrome *b* gene, the complete sequence of tRNA-Ser gene and a part of the NADH dehydrogenase subunit 1 gene. Genomic DNA was extracted from the thoraces, wings, legs and abdomens of individual sand flies using the QIAmp DNA Mini Kit (Qiagen, Germany) following the manufacturer’s instructions, and modified by crushing sand fly tissues with a piston pellet (Treff, Switzerland), and using an elution volume of 200 μl.

mtDNA amplifications were performed in a 50 μl volume using 5 μl of extracted DNA, 50 pmol of each of the primers, 10 mM of Tris HCl (pH 8.3), 1.5 mM of MgCl_2_, 50 mM of KCl, 0.01 % of Triton X 100, 200 μM of dNTP and 1.25 units of *Taq* polymerase (5 prime, Germany). The cycle profiles were marker dependent. Each PCR began by an initial denaturation step at 94 °C for 3 min and ended with a final extension at 68 °C for 10 min. Amplification of a fragment of cytochrome *b* (Cyt b) gene was done by using the primers N1N-PDR: (5′-CA(T/C) ATT CAA CC(A/T) GAA TGA TA-3′) and C3B-PDR: (5′-GGT A(C/T)(A/T) TTG CCT CGA (T/A)TT CG(T/A) TAT GA-3′) following the method previously published [10]: 5 cycles of denaturation at 94 °C for 30 s, annealing at 40 °C for 60 s and extension at 68 °C for 60 s, followed by 35 cycles of denaturation at 94 °C for 60 s, annealing at 44 °C for 60 s and extension at 68 °C for 60 s.

The D1 and D2 fragments of the 28S rDNA were amplified using the primer couple C1′: 5′-ACC CGC TGA ATT TAA GCA T-3′ and D2: 5′-TCC GTG TTT CAA GAC GGG-3′ following the thermal profile: 30 cycles with 1 min 94 °C, 1 min 58 °C, 1 min 68 °C using the primers [11]. The D8 domain of the 28S rDNA was amplified using the primers C7′ (5′-GTG CAG ATC TTG GTG GTA GT-3′) and D8E (5′-GCT TTG TTT TAA TTA AAC AGT-3′) following the thermal profile: 40 cycles of denaturation at 94 °C for 30 s, annealing at 48 °C for 40 s and extension at 68 °C for 90 s [12].

Amplicons were analysed by electrophoresis in 1.5 % agarose gel containing ethidium bromide. Direct sequencing in both directions was performed using the primers used for DNA amplification. The correction of sequences was done using the programmes Pregap and Gap included in the Staden Package [13].

### Molecular analyses

*Phlebotomus papatasi*, *Sergentomyia schwetzi* and *Sergentomyia boironis* were selected as outgroups because the phylogenetical position of the genus *Spelaeomyia* is doubtful and the selected species belong to two different genera: *Phlebotomus* and *Sergentomyia. Sergentomyia schwetzi* is widespread in Africa whereas *Se. boironis* is endemic from Madagascar. Moreover, sequences for these species were available for each molecular marker.

Sequence alignments were performed using the ClustalW routine included in the Bioedit software [14] and checked “by eye” in order to respect the three following criteria: (i) minimize the number of inferred mutations (number of steps); (ii) prefer substitution to insertion-deletion; and (iii) prefer transitions over transversions, because they have a higher probability of occurrence [15–17].

The molecular datasets were analysed by Bayesian methods with MrBayes 3.2 [18]. Trees were rooted with a sequence for *Ph. papatasi* (specimen code Ph-papatasi-HM992927) as outgroup. One partition for each codon position of the mitochondrial gene (Cyt b) and one partition for the nuclear genes D1D2 and D8 were implemented to explore the best substitution model, individually and concatenated. The best-fit models of nucleotide substitutions were selected with jModelTest v2.1.4 [19] using the Akaike Information Criterion (AIC). Bayesian analyses were carried out independently for each gene and for the concatenated data (2 million generations, saving trees every 100 generations). The first 25 % of the generated trees were discarded as 'burn-in’. The robustness of trees nodes was assessed by clade posterior probability values (CPP).

## Results

### Molecular analyses

The origin of the specimens of this study and their accession numbers are listed in Table 1.

**Table 1:** Species samples studied here with data on the origin, biotope and year of collection

Species	Country	Area	Biotope	Year of collection	Gender	Specimen	GenBank accession numbers		
							D1D2 rDNA	D8 rDNA	Cyt b mtDNA
*Sa. mirabilis*	Republic	Mayama	Meya-N’Zouari Caves	1987	♂	MIRA1	KU564275	KU555395	KT266679
	of the Congo				♀	MIRA2	KU564276	KU555396	KT266680
					♀	MIRA4	KU564277	KU555397	KT266681
	Gabon	La Lopé National Park, Mikongo	Rainforest	2013	♀	MIK26	KU564278	KU555398	KT266682
*Sa. darlingi*	Mali	Bandiagara	Underground house	2010	♀	M20	KU564272	KU555392	KT266677
					♀	M21	KU564273	KU555393	KT266678
					♂	M60	KU564274	KU555394	KU555391
*Sa. emilii*	Gabon	Djibilong cave, La Lékabi Ranch	Open cave	2010	♂	EMIL1	KU564279	KU555399	KT266675
					♂	EMIL2	KU564280	KU555400	KT266676
*Sa. moucheti*	Gabon	La Lopé National Park, Mikongo	Rainforest	2013	♂	GAB27	KU564281	KU555401	KT266683
		Lékédi private park Bakoumba	Rainforest	2013	♂	GAB65	KU564282	KU555402	KT266684
					♀	GAB71	KU564283	KU555403	KT266685
					♀	GAB84	KU564284	KU555404	KT266686
*Se.schwetzi*	Algeria	Tamanrasset		2006		*Se.schwetzi*	KU564286	KU555406	KU564287

We performed three analyses: the first with the mitochondrial gene with 402 aligned base pairs (bp), the second with all 1,266 bp nuclear genes (662 for D1D2 and 604 for D8) and a third combined-data analysis of all the molecular data with 1,668 bp.

The symmetrical model with a gamma distributed among-site variation (SYM + G) was indicated as the best-fit model for the mitochondrial cytochrome *b* gene. The general time reversible model with gamma distributed among-site rate variation (GTR + G) was indicated as the best-fit model for both separated and concatenated 28S rDNA markers (D1, D2 and D8). The GTR + G + I model was indicated as the best-fit model for concatenated mtDNA and rDNA.

**Fig. 1:**
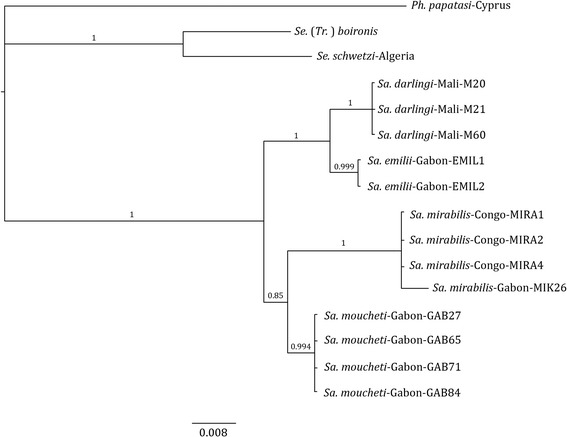
Bayesian tree resulting from the phylogenetic analysis of the concatenated dataset from rDNA. The tree is rooted with one sequence of *Ph. papatasi* (specimen code *Ph. papatasi*-HM992927). Robustness of nodes is indicated by the Clade Posterior Probability values (CPP)

**Fig. 2:**
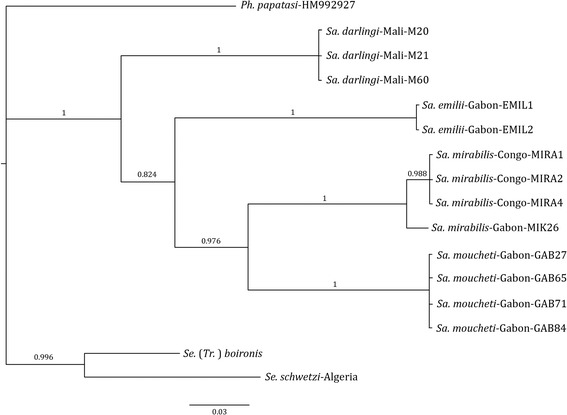
Bayesian tree resulting from the phylogenetic analysis CytB dataset. The tree is rooted with one sequence of *Ph. papatasi* (specimen code *Ph. papatasi*-HM992927). Robustness of nodes is indicated by the Clade Posterior Probability values (CPP)

**Fig. 3:**
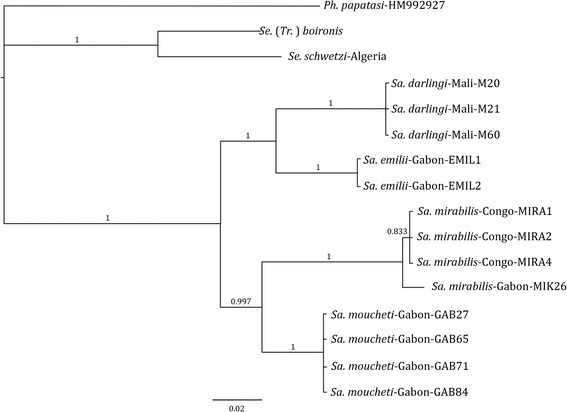
Bayesian tree resulting from the phylogenetic analysis of the combined dataset from rDNA and mtDNA. The tree is rooted with one sequence of *Ph. papatasi* (specimen code *Ph. papatasi*-HM992927). Robustness of nodes is indicated by the Clade Posterior Probability values (CPP)

Trees from Bayesian inference analyses of the concatenated-data, mtDNA, and rDNA are summarized in Figs. 1, 2 and 3. Most nodes of the topologies obtained from concatenated genes (rDNA and Cyt b) are well supported with CPP values comprised between 83 and 100 %. Based on the analyses on both, the rDNA and Cyt b genes, *Sa. mirabilis* and *Sa. moucheti* appeared as sister species. However, the positioning of *Sa. emilii* remained problematic. At first, based on rDNA markers (Fig. 1), *Sa. darlingi* and *Sa. emilii* appeared to be sister species. However, in the Cyt b sequences, *Sa. darlingi* clearly diverged and *Sa. emilii* ultimately appeared as the sister group of *Sa. mirabilis* and *Sa. moucheti* (Fig. 2). Nevertheless, conflict of these discordant nodes is strongly supported by both data types. This strongly supported conflict is resolved in favour of the rDNA in the combined-data tree (Fig. 3).

The newly-generated sequences from the males and females of *Sa. moucheti* were identical for each marker.

### Description of the male of *Spelaeomyia moucheti*

***Locality*:** La Lopé National Park, Mikongo and Lékédi private park, Bakoumba, Gabon 2013, CDC Light trap.

***Voucher material*:** MIK2, MIK35, MIK141, GAB27, GAB65, and GAB88.

[Measurements based on six specimens; minimum and maximum values are indicated.]

**Fig. 4:**
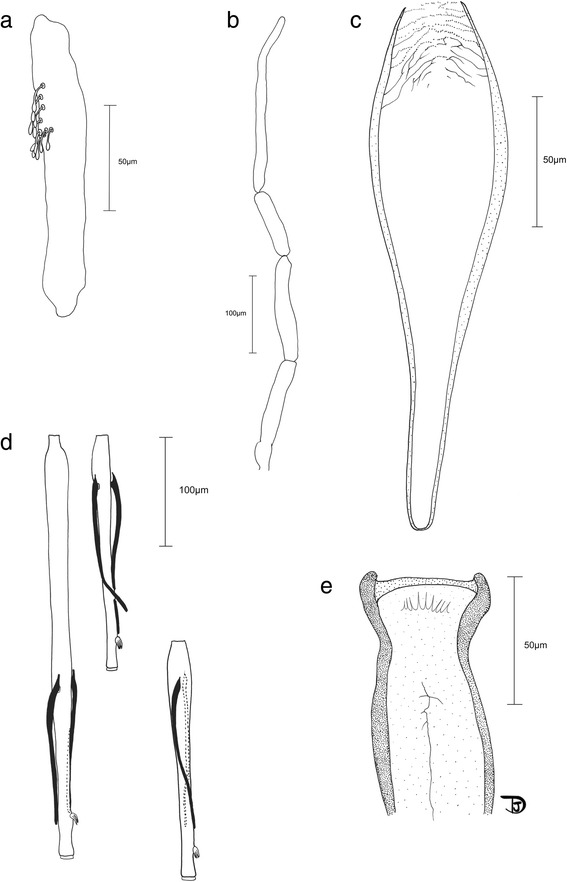
Drawings of the male of *Spelaeomyia moucheti*, head parts. **a** Third palpal segment exhibiting Newstead spines. **b** Palp. **c** Pharynx. **d** Antennal segments III to V (= flagellomeres I to III). **e** Cibarium

*Head* (Fig. 4): Interocular suture incomplete. Cibarium with 8–10 teeth arranged on concave arc directed backwards and without denticles. Pigmented patch absent. Pharynx fine, *c.*200 long, slightly shrunk backwards. Pharyngeal armature not well developed, with some wrinkles and very small denticles. Palpal formula 1-4-2-3-5; third palpal article with about 10 club-like Newstead spines. Ascoid formula: 2/III-XV; ascoids rather long but not going over next articulation; base of ascoid pointing backwards (Fig. 4d). A III (= flagellomere I) 372–385, A IV (= flagellomere II) 222–225, A V (= flagellomere III) 227, A III < A IV + A V; labrum 215–240 μm. A III/labrum = 1.60–1.73. Labial furca closed.

**Fig. 5:**
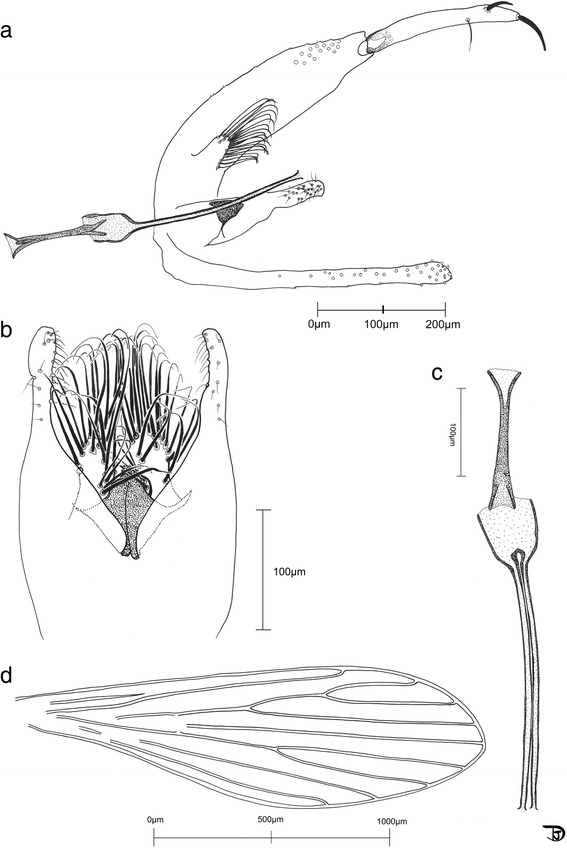
Drawings of the male of *Spelaeomyia moucheti*, genitalia and wing. **a** Genitalia, total view. **b** Ventral view of the genitalia showing the aedeagus, the parameres and the basal lobes of the coxites. **c** Genital pump and whole ducts. **d** Wing

*Thorax* (Fig. 5d): Wing length 1,998–2,140, width 560–600, length/width ratio 3.55–3.57; α = 432–452; β = 318–328; δ = 95–100; ɣ = 250–300; wing width/ɣ 2.0–2.24; π = 350–360.

*Genitalia* (Fig. 5): Coxite 414–433 long, bearing a basal lobe on its internal face; basal lobe with about twenty strong setae. Coxite/lateral lobe ratio 7.4–7.9. Style rather long and straight, reducing apically, 247–256, bearing two spines: one terminal and one subterminal. One seta at last third of style present. Paramere simple, 210–240 in total length. Surstyle 449–477 long. Penis short, 47–56, triangular, heavily sclerotized. Genital pump heavily sclerotized, well developed, 194–212. Genital ducts short, 253–286, relatively wide, enlarged at the top.

### Updates on the morphology of the female of *Spelaeomyia moucheti*

**Fig. 6:**
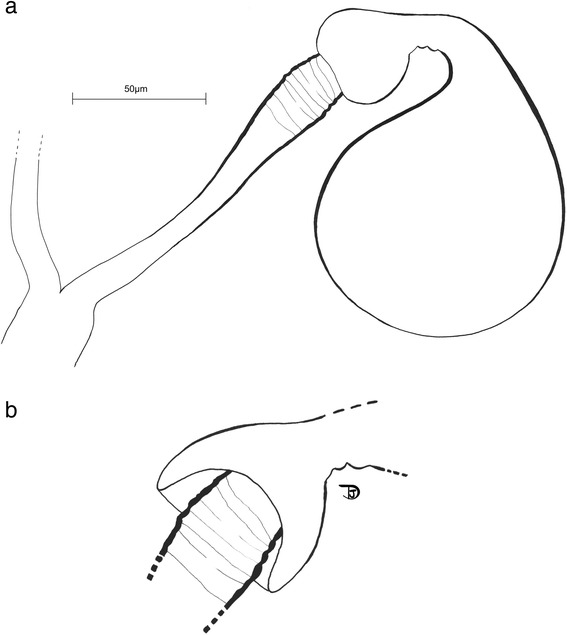
Drawings of the female of *Spelaeomyia moucheti*, spermathecae. **a** Spermathecae, total view. **b** Detail of bulbous process of the spermathecae overlapping the apex of the spermathecal duct after Euparal mounting (same specimen)

It appeared important to us to make a new illustration (Fig. 6a) of the female spermathecae to update those from the original description [20]. The structure of the spermathecae was elucidated due to the application of Marc-André clearing. The spermathecal ducts are fused at their base and the walls of the spermathecae are more sclerotized on the sides than on the apex. Herein we also describe a new morphological feature on the spermathecal ducts that are slightly striate at the apex, just before the bulbous process of the spermathecae. In Fig. 6b the bulbous process of the spermathecae overlapping the apex of the spermathecal duct is illustrated (specimen mounted in Euparal).

### Updates on the ecology of samples collected in Gabon

Genus *Spelaeomyia* is known to be strictly cavernicolous, but in Gabon we collected *Sa. mirabilis* only in the rainforest, *Sa. moucheti*, in both habitats (but in greater number outside caves) and *Sa. emilii* only in caves.

## Discussion

*Spelaeomyia* has been defined as a group of cavernicolous sand flies, according to the following characters [1]: style with two spines of which one is terminal and with a small seta; process with long hairs near the base of the coxite; penis sheet rudimentary; genital filaments short and thick with terminal process; legs very long; cibarial armature consisting of a row of long pointed teeth; pharynx unarmed; spermathecae irregularly crinkled sack.

Lewis & Kirk [21] redefined later the genus *Spelaeomyia* as follows: several erect hairs on the second to sixth abdominal tergites; style with one or two spines and a small seta; a paired process, with short hairs, and a median process between bases of coxites; penis sheath of unusual shape, either very short and blunt or very long and pointed; penis filament with a flat hyaline process near tip; legs very long; buccal cavity with no more than 14 principal teeth some of them widely separated; a sensory papilla on antennal segments III, IV and V; pharynx unarmed; spermathecae irregularly crinkled sack.

This definition needs to be modified: (i) the males of *Sa. darlingi* and *Sa. emilii* exhibit only one distal spine on the style and their coxal setae cannot be considered as a spine in our opinion; (ii) *Sa. darlingi* has long genital filaments; (iii) the penis of *Sa. darlingi* is long and pointed. Moreover, the females are characterized by their sensorial trough (translation of the French “*dépression sensorielle*” used by French-speaking authors observing this structure) at the distal part of the abdomen, first observed in *Sa. mirabilis* [22] and then confirmed in all species of the genus *Spelaeomyia* [7]. The ecological data also need to be modified. Based on literature [7, 22–24], the genus *Spelaeomyia* is strictly cavernicolous. In our prospections in Gabon, *Sa. emilii* was indeed exclusively collected in caves. However, *Sa. mirabilis* was only recorded in rainforest biotopes. *Spelaeomyia moucheti* were found in caves but they were mainly collected in rainforests. Therefore, it is wrong to consider *Sa. mirabilis* and *Sa. moucheti* as strictly cavernicolous species.

The unknown males are considered and described as those of *Sa. moucheti* because: (i) they were captured with females of *Sa. moucheti*, the same day, in the same traps; (ii) both belong to the same genus, and we suppose, to the same species; and (iii) the mtDNA cytochrome *b*, rDNA D1D2 and D8 sequences obtained from these males and from *Sa. moucheti* females are exactly similar. With the description of the male of *Sa. moucheti*, we propose an identification key to the species of *Spelaeomyia* based on male morphology:

1. Style with one spine (terminal) ........................................ 2
  Style with at least two spines (one terminal, one subterminal) ....................................................................... 3
2. Paramere simple, blunt ending; coxal setae directly on the coxite; genital filaments short .................... *Sa. emilii*
  Paramere rounded at the top, with a superior finger-like process at the middle; coxal setae on a long process; genital filaments long .......................................... *Sa. darlingi*
3. Paramere with a tuft of grouped setae at the top; genital ducts with a process at the end ..................... *Sa. mirabilis*
  No tuft of setae at the top of the paramere; genital ducts without a process but apically widened .............................................................................. *Sa. moucheti*

We strongly consider *Sa. moucheti* as the sister species of *Sa. mirabilis* whereas the position of *Sa. darlingi* and *Sa. emilii* as sister species was not resolved. If we look for morphological characters, *Sa. moucheti* and *Sa. mirabilis* share long ascoids reaching the next articulation and exhibit two spines on the style and a short penis. Here, the morphological characters are in agreement with the results from analyses of molecular markers. *Spelaeomyia darlingi* and *Sa. emilii* share only one spine on the style and have relatively short ascoids.

Strongly supported conflicts (for which conflicting clades are strongly supported by each type of data) tend to be uncommon and may be resolved in favor of either mtDNA or nucDNA in almost equal frequency. Combined analyses of mtDNA and nucDNA are common [25], but the consequences of combining these data are largely unexplored. This trend is somewhat unsettling given that the use of mtDNA is somewhat controversial, and given the possibility that mtDNA might dominate combined analyses due to larger numbers of variable characters [25].

## Conclusions

The description of the male of *Sa. moucheti* is proposed and supported by molecular homologies with females. An update on the morphology of the female of *Sa. moucheti* is made and advice is given on the observation of spermathecae. Molecular and morphological data showed that *Sa. moucheti* is closely related to *Sa. mirabilis*. A new identification key for species of *Spelaeomyia* is proposed based on male morphology. New ecological data suggest that the genus *Spelaeomyia* is not strictly cavernicolous and some of its members are adapted to various environments. Additional studies regarding taxa in sand flies, particularly for the species of the genus *Spelaeomyia*, remain necessary. A study of the consequences of combining nuclear and mitochondrial data for phylogenetic analysis would be required as well.

## Abbreviations

CIRMF, Centre International de Recherches Médicales de Franceville; Cyt b, in the manuscript, we call “Cyt b” a part of the cytochrome *b* gene, the complete sequence of tRNA-Ser gene and a part of the NADH dehydrogenase subunit 1 gene; IRD, Institut de Recherche pour le Développement; *Sa, Spelaeomyia; Se, Sergentomyia*
